# Oxygen Enrichment Membranes for Kuwait Power Plants: A Case Study

**DOI:** 10.3390/membranes11030211

**Published:** 2021-03-17

**Authors:** Yousef Alqaheem, Fajer Alswaileh

**Affiliations:** Petroleum Research Center, Kuwait Institute for Scientific Research, P.O. Box 24885, Safat 13109, Kuwait; fswaileh@kisr.edu.kw

**Keywords:** enriched oxygen, gas-separation membrane, fuel combustion, carbon dioxide emissions, power plant

## Abstract

Power plants are considered as the major source of carbon dioxide pollution in Kuwait. The gas is released from the combustion of fuel with air to convert water into steam. It has been proven that the use of enriched oxygen can reduce fuel consumption and minimize emissions. In this study, UniSim (Honeywell, Charlotte, NC, USA) was used to estimate the fuel savings and carbon dioxide emissions of the largest power plant in Kuwait (Alzour). Results showed that at 30 mol% oxygen, the fuel consumption was lowered by 8%, with a reduction in carbon dioxide emissions by 3524 tons per day. An economic analysis was performed on the use of a membrane unit to produce 30 mol% oxygen. At current market prices, the unit is not economical. However, the system can achieve a payback duration of 3 years if natural gas price increases to USD 6.74 or the compressor cost decreases to USD 52 per kW. Currently, the research and development sector is targeting a membrane fabrication cost of less than USD 10 per m^2^ to make the membrane process more attractive.

## 1. Introduction

**Figure 1:**
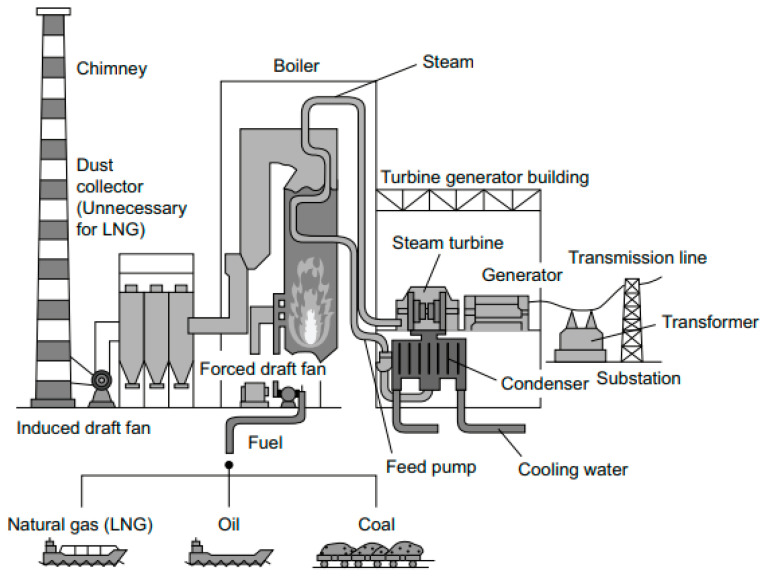
Power generation from the combustion of fossil fuel [7].

**Figure 2:**
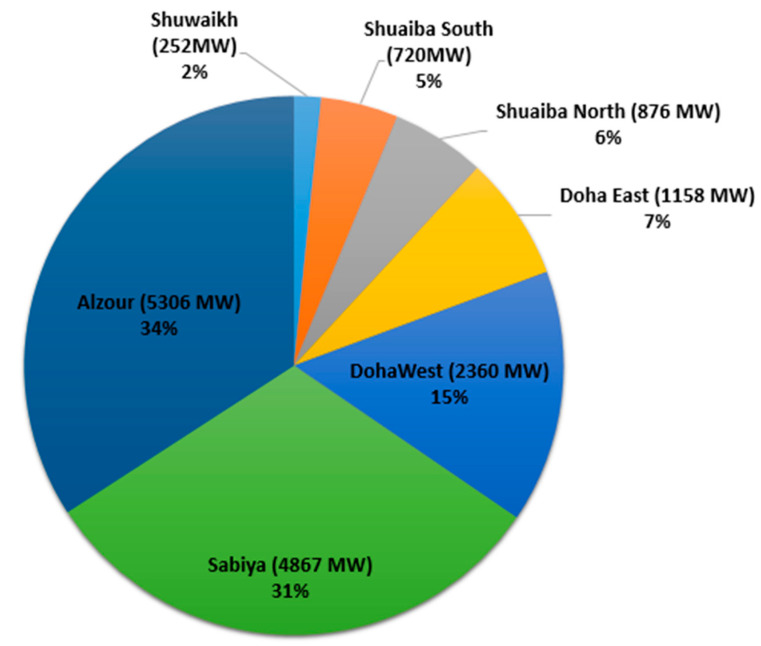
The seven power stations in Kuwait and their output power.

Carbon dioxide is one of the greenhouse gases that can cause global warming. Greenhouse gases absorb solar radiation, resulting in an increase in climate temperature [1]. In Kuwait, there are many sources of carbon dioxide, such as vehicles, refineries, and power plants. Nevertheless, it has been reported that power plants account for 42% of the total released carbon dioxide [2]. The gas is produced in the power plant from the combustion reaction of air with fuel to provide the heat to convert water into steam. The steam then drives a turbine to generate electricity, as shown in Figure 1. There are seven power stations in Kuwait, and Alzour is the largest station with a power output of 5300 MW (Figure 2). The station was commissioned in 1987 and it consists of eight steam turbines and four gas turbines [3]. This station alone produces 30% of the total emitted carbon dioxide from power plants.

In power plants, fuel is usually combusted with air. However, air contains only 21 mol% of oxygen. It has been proven that the use of enriched oxygen of more than 21 mol% can increase combustion efficiency [4]. This is because the enriched oxygen has lower nitrogen content, which increases the flame temperature and reduces the formation of unwanted nitrogen oxides [5]. Furthermore, the concentrated oxygen minimizes incomplete combustion, resulting in a lower production of carbon monoxide [6].

There are mainly three commercialized technologies for the production of oxygen streams: Cryogenic distillation, pressure swing adsorption (PSA), and membrane. Cryogenic distillation is the earliest technology for oxygen purification. It is based on cooling the air to an extreme temperature of −175 °C, which causes the oxygen to liquefy before nitrogen [8]. In PSA, air enters a zeolite bed in which nitrogen is adsorbed and oxygen passes [9]. With time, the bed becomes saturated with nitrogen, and a regeneration step is required by either reducing the operating pressure or increasing the temperature [10]. The membrane, on the other hand, works based on the solution diffusion model in which the gas is adsorbed on the polymer surface. The gas then diffuses through the polymer by the mean of free volumes, which acts as selective voids [11]. Comparing the three technologies, cryogenic distillation has been proven to provide the highest oxygen purity (up to 99.999%), while the membrane is known to provide the best economical solution at the expense of oxygen purity [12,13]. Yet, for most industrial applications, it is reported that the use of 30 mol% of oxygen is sufficient as it does not require a major process modification (due to safety issues). This means that the membrane is the best choice for the generation of 30 mol% oxygen in terms of capital and operating costs, as shown in Table 1. Nevertheless, it should be noted that the membrane performance might decrease with time due to the accumulation of the gas in the boundary layer [14,15,16]. This phenomenon is known as concentration polarization and can be reduced by increasing the feed gas velocity [17].

In this paper, UniSim (Honeywell, Charlotte, North Carolina, USA) was used to simulate the performance of the Alzour power station using natural gas as a fuel and air as a source of oxygen. The software is widely used in chemical plants and refineries to perform material and energy balances for better optimization and maximizing profits. The simulation was run using different oxygen concentrations, and the fuel savings were calculated. The simulation was also utilized to monitor carbon dioxide content in the flue gas. An economic study was also performed for the installation of a membrane unit to provide the enriched oxygen for the furnace, and the net savings were estimated.

## 2. Methodology

In Kuwait, various fossil fuels are used in power plants such as natural gas, fuel oil, gas oil, and crude. However, the highest fuel consumption is for natural gas [2]. Therefore, in this study, the fuel was assumed to be fully natural gas with a chemical composition given in Table 2. UniSim (Honeywell, Charlotte, NC, USA) was used to simulate the performance of the Alzour power plant to produce a power of 5300 MW. The operating conditions are given in Table 3. Water was fed at 34 °C (140 bar) and heated to 450 °C by the flue gas generated from the combustion of air with natural gas. The oxygen concentration of the feed gas was varied from 21 mol% to 100 mol%, and air-to-fuel ratio was calculated based on a 2 mol% oxygen excess in the flue gas. The combustion was modeled using the integrated furnace unit in UniSim. The simulation software does not include a membrane unit to produce enriched oxygen. Thus, the system was simulated using a spreadsheet in which mass balance was performed across the membrane:

(1) $$x_{F}n_{F}=y_{P}n_{P}+x_{R}n_{R}$$

where *n* is the number of moles, $x_{F}$ is the mole fraction of component *i* in the feed, $x_{R}$ is the mole fraction in the retentate (vented stream), and $y_{P}$ is the mole fraction in the permeate (product oxygen). Equation (1) can be rewritten as:

(2) $$y_{P}n_{P}=x_{F}n_{F}-x_{R}n_{R}=QA\left(\bar{xP_{F}-yP_{P}}\right)$$

where *Q* is the permeance, *A* is the membrane area, and the last term is the trans-membrane pressure difference, which can be simplified to [21]:

(3) $$\left(\bar{xP_{F}-yP_{P}}\right)≅{\left[x_{F}x_{R}\left(\frac{x_{F}+x_{R}}{2}\right)\right]}^{\frac{1}{3}}$$

where *P_F_* is the feed pressure and *P_P_* is the permeate pressure. To solve the above equations, the stage cut (*θ*) was guessed using:

(4) $$\theta_{Guess}=\frac{y_{P}n_{P}}{x_{F}n_{F}}$$

After that, the following equations were solved simultaneously to calculate $x_{R}$ and $y_{P}$:

(5) $$y_{P}n_{P}=x_{F}n_{F}-x_{R}n_{R}$$

(6) $$\overset{n}{\underset{i=1}{\sum }}x_{F}=1;\;\overset{n}{\underset{i=1}{\sum }}x_{R}=1;\;\overset{n}{\underset{i=1}{\sum }}y_{P}=1$$

Thereafter, the stage cut was recalculated and compared with the guessed value using the “adjust function” in UniSim until the guess value was equal to the calculated one with a tolerance of 0.00001:

(7) $$\theta_{Calc}=\frac{QA\left(\bar{xP_{F}-yP_{P}}\right)}{x_{F}n_{F}}$$

The operating conditions of the feed, retentate, and permeate are given in Table 4. Oxygen permeance of 1200 GPU represents the performance of commercial perfluoropolymer membranes. The membranes are known to have an oxygen-to-nitrogen selectivity of 4 [6].

## 3. Results and Discussion

### 3.1. Fuel Savings

**Figure 3:**
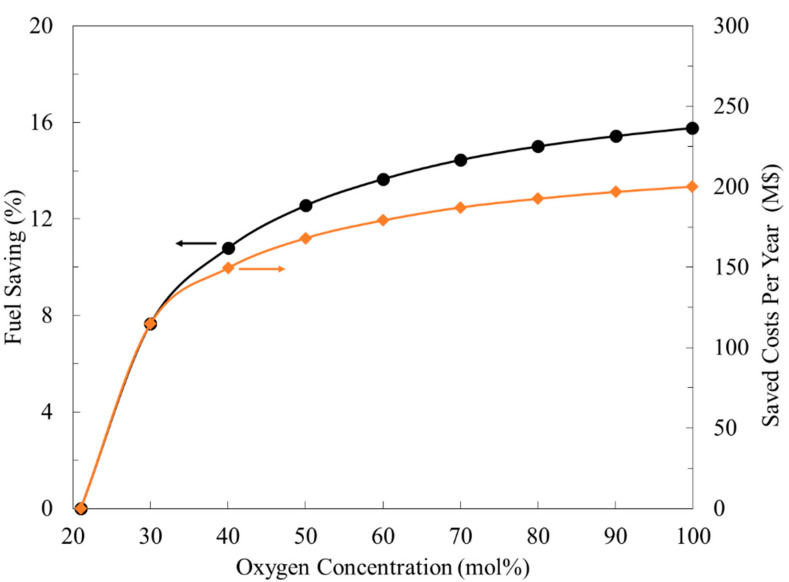
Enriched oxygen concentration and the corresponding natural gas fuel savings.

The oxygen concentration in the feed versus fuel saving is given in Figure 3. The oxygen enrichment changes exponentially bounded with fuel reduction. The significant saving region occurred when over 21 mol% to 40 mol% of oxygen was used. For example, at 30 mol% oxygen, the fuel consumption was lowered by 8% (savings of USD 115 million), while at 40 mol% oxygen, the fuel was decreased by 11% (savings of USD 150 million). However, for a pure oxygen stream, only a reduction of 16% was noticed. The savings were calculated based on the current natural gas price of USD 2.9.

### 3.2. Carbon Dioxide Emissions

**Figure 4:**
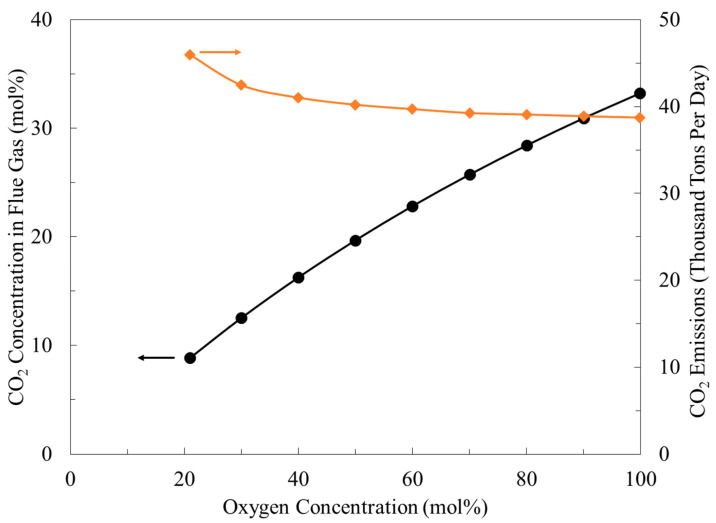
Carbon dioxide concentration in the flue gas and the reduction in emissions due to the use of enriched oxygen.

The data of carbon dioxide content in the flue gas against oxygen enrichment are given in Figure 4. The concentration of carbon dioxide changed linearly with oxygen enrichment. For instance, at 30 mol% oxygen, the concentration of carbon dioxide was 12%, while at 100 mol% oxygen, the content increased significantly to 33%. It is expected from these figures that carbon dioxide emissions were increased as well due to the increase in carbon dioxide concentration. However, Figure 4 shows the opposite, and carbon dioxide emissions (in tons per day) were decreased. For example, when air was used, the carbon dioxide discharge was 45,987 tons per day, but if 30 mol% of oxygen was used, the emissions would have been minimized to 42,462 tons per day. The use of pure oxygen can further reduce carbon dioxide to 38,736 tons per day. The decrease in carbon dioxide emissions can be interpreted by the reduction in the air-to-fuel ratio [22].

### 3.3. Economic Assessment

**Figure 5:**
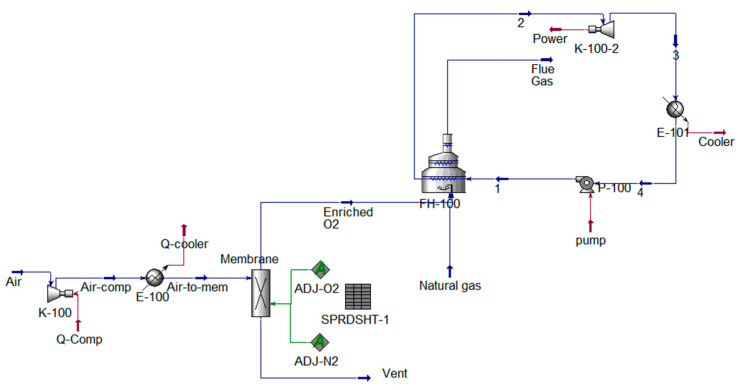
UniSim simulation of the Alzour power plant using enriched oxygen of 30 mol% by a membrane unit.

Based on the above results and because enriched oxygen of 30 mol% does not require a major process modification, this stream was selected to perform the economic analysis. First, the required membrane area and the compressor input power were determined. Figure 5 shows the process flow diagram for the production of enriched oxygen (30 mol%) to generate an electric power of 5300 MW.

The results show that the required membrane area is 3,500,000 m^2^, with a compressor input power of 2228 MW. This power requirement is massive, and it accounts for 42% of the total output power of Alzour power plant. This extensive energy is related to the large volume of the feed air of 32 million m^3^ h^−1^. 

Economic evaluation to produce 30 mol% oxygen for the Alzour power plant is performed in Table 5. The current membrane skid price is USD 50 per m^2^, while the compressor cost is USD 200 per kW [6]. This gives a membrane capital cost of USD 175 million, with an additional compressor cost of USD 446 million. Therefore, the total capital cost of the system is USD 621 million. Annual operating costs are based on the maintenance cost (assumed 5% of the capital cost) and electricity bill (to run the compressor). The electricity tariff for industrial activities in Kuwait is USD 0.0015 per kWh. This gives a total annual operating cost for the membrane system of USD 60 million. At 30 mol% oxygen, the fuel saving is USD 115 million but the capital and operating costs are USD 681 million. This gives a negative net saving of USD—566 million for the first year. In the following year, the capital cost is needed, and thus, the saving is USD 55 million However, this amount is used to recover the negative net in the first year. This means that 12 years are needed to fully recover the costs of the membrane system (payback period). Nevertheless, this statement is not true because, after 5 years, the membrane needs to be replaced, which costs USD 25 per m^2^ [6]. Furthermore, the compressor life is expected to be between 10 to 15 years. So, at the current prices, the membrane is considered uneconomical.

There are three parameters that can greatly affect the feasibility of using the membrane system: The natural gas price and the capital cost of the membrane skid and the compressor. Usually, a 3-year payback period is acceptable for most industries [24]. The scenarios in which the membrane is profitable are given in Table 6. In scenario 1, the capital cost of the membrane and compressor were held while the price of natural gas was raised. For the process to be economical, the price of fuel should increase from USD 2.9 to USD 6.74. In scenario 2, the natural gas price and the membrane skid cost remained constant at current prices, but the cost of the compressor was lowered. A maximum price of the compressor of USD 52 per kW is required for the process to be feasible. In scenario 3, the cost of the membrane skid was minimized (keeping natural gas and compressor costs constant) but it was not economical due to the short payback duration of 3 years. If the membrane skid price was decreased to USD 10 (research and development target), a payback duration of 8 years would be needed. In the last scenario, the fuel price was maintained at the current value, but the costs of the membrane skid and compressor were reduced. It was found that, if the membrane skid price was USD 10 and the compressor cost was USD 87.6 per kW, then the process would be profitable.

In this study, 1.28 million tons of carbon dioxide from the Alzour power plant were reduced annually, using the membrane system, for a total investment of USD 565 million. Another method to lower the emissions is by capturing carbon dioxide in the flue gas. It has been estimated that the capturing carbon dioxide will cost around USD 100 per ton using PSA technology [25]. The expected carbon dioxide selling price is USD 20 per ton [26]. This means that a net investment of USD 103 million is needed to capture 1.28 million tons of carbon dioxide. Therefore, for this study, carbon dioxide capture is a better option than using the oxy-fuel combustion.

## 4. Conclusions

Power plants are the major source of carbon dioxide pollution in Kuwait. One of the solutions to reduce emissions is using enriched oxygen for fuel combustion. Research has proven that the concentrated oxygen can reduce fuel consumption as well. In this study, the largest power station in Kuwait (Alzour) was simulated in UniSim to produce 5300 MW of output power. The oxygen content was varied in the feed from 21 mol% to 100 mol% and the fuel savings, along with the carbon dioxide content in the flue gas, were calculated. The results show the fuel saving increases exponentially bounded with the oxygen content. Enriched oxygen with 30 mol% gave a fuel saving of 8% with a reduction in carbon dioxide by 3525 tons per day. The membrane system was simulated in UniSim (Honeywell, Charlotte, NC, USA) to determine the membrane area and the compressor power to perform the economical assessment. The study shows that the membrane process requires 12 years to fully recover the capital and operating costs, which is not feasible due to the additional costs for membrane replacement and the compressor. For the process to be profitable with a payback duration of three years, natural gas price should increase to USD 6.74 or the compressor cost should be reduced to USD 52 dollars per kW. It was also found that, for this study, post-combustion carbon dioxide capture provides a better choice for reduction of carbon emissions due to the lower investment.

## Figures and Tables

**Table 1:** Capital and operating costs for producing 30 mol% oxygen by different technologies [13,18,19].

Technology	Capital Cost (USD/tons O_2_)	Operating Cost (USD/tons O_2_)
Cryogenic Distillation	25,000–525,000	39
Pressure Swing Adsorption (PSA)	13,000–70,000	26
Membrane	11,000–27,000	23

**Table 2:** Composition of Kuwait natural gas used in this study [20].

Component	Mole Fraction (%)
Methane (CH_4_)	87
Ethane (C_2_H_6_)	9
Propane (C_3_H_8_)	2
Butane (C_4_H_10_)	1
Nitrogen (N_2_)	1

**Table 3:** Operating conditions for the simulation of the Alzour power plant.

Property	Condition
Water	
Inlet water temperature	34 °C
Steam temperature	450 °C
Steam pressure	140 bar
Air	
Air pressure	1 bar
Air temperature	25 °C
Oxygen concentration	21–100 mol%
Natural Gas	
Natural gas pressure	1 bar
Natural gas temperature	25 °C
Furnace	
Flue gas temperature	450 °C
Flue gas excess oxygen	2 mol%
Turbine	
Turbine Power	5300 MW
Outlet pressure	0.05 bar

**Table 4:** Operating conditions of the membrane unit for oxygen enrichment.

Property	Condition
Feed pressure	4 bar
Feed temperature	25 °C
Retentate pressure	4 bar
Retentate temperature	25 °C
Permeate pressure	1 bar
Oxygen permeance	1200 GPU *
Nitrogen permeance	400 GPU *

* Reference [6].

**Table 5:** Economic analysis to produce 30 mol% oxygen for the Alzour power plant [6,23].

Parameter	Value
Capital Cost ^1^	
Membrane skid	USD 50 per m^2^
Membrane area	3,500,000 m^2^
Membrane cost	USD 175,000,000
Compressor price	USD 200 per kW
Compressor power	2,228,000 kW
Compressor cost	USD 445,600,000
Total capital cost	USD 620,600,000
Annual Operating Cost ^2^	
Electricity Bill	USD 0.0015 per kWh
Maintenance	5% capital cost
Total Operating Cost	USD 60,305,920
Net Savings (First Year)	
Natural gas price	USD 2.9 per MMBTU *
Annual fuel saving	39,647,894 MMBTU
Saving	USD 114,978,893
Net savings	USD—565,927,027

^1^ Coolers’ costs are not included. ^2^ Labor cost is not covered. * MMBTU: one million British thermal units.

**Table 6:** Scenarios in which the membrane system could be profitable for oxygen enrichment (30 mol%) at the Alzour power plant for a payback duration of 3 years.

Parameter	Target Value	Current Value
Scenario 1 (natural gas price)		
Natural gas price	USD 6.74 per MMBTU	USD 2.9 per MMBTU
Compressor cost	-	USD 200 per KW
Membrane skid cost	-	USD 50 per m^2^
Scenario 2 (compressor cost)		
Natural gas price	-	USD 2.9 per MMBTU
Compressor cost	USD 52 per kW	USD 200 per kW
Membrane skid cost	-	USD 50 per m^2^
Scenario 3 (membrane skid cost)		
Natural gas price	-	USD 2.9 per MMBTU
Compressor cost	-	USD 200 per kW
Membrane skid cost	USD 10 (8 years payback)	USD 50 per m^2^
Scenario 4 (compressor and membrane costs)		
Natural gas price	-	USD 2.9 per MMBTU
Compressor cost	USD 87.6 per kW	USD 200 per kW
Membrane skid cost	USD 10 per m^2^	USD 50 per m^2^

## Data Availability

Derived data supporting the findings of this study are available from the corresponding author on request.
